# ­­­­Evaluation of Design and Fabrication of Food-Grade Nanofibers from Chitosan-Gelatin for Nanoencapsulation of Stigmasterol Using the Electrospinning Method

**DOI:** 10.34172/apb.2021.059

**Published:** 2020-08-24

**Authors:** Mir-Michael Mousavi, Mohammadali Torbati, Parastou Farshi, Hedayat Hosseini, Masoud Aman Mohammadi, Seyede Marzieh Hosseini, Simzar Hosseinzadeh

**Affiliations:** ^1^(Department) National Nutrition and Food Technology Research Institute, Faculty of Nutrition Sciences and Food Technology, Shahid Beheshti University of Medical Sciences, Tehran, Iran.; ^2^Students’ Research Committee, National Nutrition and Food Technology Research Institute, Faculty of Nutrition Sciences and Food Technology, Shahid Beheshti University of Medical Sciences, Tehran, Iran.; ^3^Department of Food Science and Technology, Faculty of Nutrition and Food Sciences, Nutrition Research Center, Tabriz University of Medical Sciences, Tabriz, Iran.; ^4^Food Science Institute, Kansas State University, Manhattan, KS, USA.; ^5^Department of Food Science and Technology, National Nutrition and Food Technology Research Institute, Faculty of Nutrition Sciences and Food Technology, Shahid Beheshti University of Medical Sciences, Tehran, Iran.; ^6^School of Advanced Technologies in Medicine, Shahid Beheshti University of Medical Sciences, Tehran, Iran.

**Keywords:** Electrospinning, Chitosan, Gelatin, Nanofibers, Stigmasterol

## Abstract

***Purpose:*** In this research, electrospinning method was employed to fabricate food-grade nanofibers (NFs) from chitosan-gelatin combination for stigmasterol encapsulation. The spinnability of mixed chitosan and gelatin solutions was investigated at different polymer ratios, and the physicochemical properties of the NFs were evaluated.

***Methods:*** The mixture solution of chitosan (1.5 % w/v) and gelatin (20 % w/v) in acetic acid indicated spinnability under the following conditions: the ratio of 25:75, voltage of 17 kV, and 15 cm capillary collector distance with a flow rate of 0.2 mL/min. Stigmasterol (0.04 % w/v) was incorporated into NFs of chitosan-gelatin at a respective ratio of 25:75.

***Results:*** Encapsulation efficiency (EE) of loaded stigmasterol was found to be 87 ± 5 %. The antioxidant ability of loaded stigmasterol was considerably higher than that observed for free stigmasterol. Scanning electron microscopy (SEM) results demonstrated the formation of the ultrathin fibers with no bead (with diameters of 217 ± 43 nm). The concentration of polymeric solution and viscosity had a notable effect on the electrospinning efficiency of the chitosan-gelatin-based NFs. The thermal stability of chitosan and gelatin fibers was more than that of native gelatin and chitosan. The in vitro stigmasterol release from these NFs followed a controlled-release pattern. The released phytosterol from chitosan formula was less than from those without chitosan formula (46 ± 3 % and 96 ± 4 % respectively).

***Conclusion:*** The obtained results suggested that gelatin had a high potential for enhancing the spinnability of chitosan under acidic conditions at optimized concentrations.

## Introduction

Phytosterols (such as stigmasterol) are bioactive compounds of vegetables which have many biological activities. The chemical structure of phytosterols consists of three rings of six-carbon atoms, one ring of five-carbon atoms, and a 3-β-hydroxyl group linked to an aliphatic chain. Phytosterols have diverse beneficial effects on human health such as reducing the cholesterol serum levels, anti-oxidative effects,^1^ anti-inflammatory effects, anti-atherosclerotic properties, and anti-cancer activities^2-4^ such as the ability to inhibit the development of colon cancer.^5^ However, water insolubility, instability, high melting temperature, chalky taste, susceptibility to oxidation, and poor absorption by the human body are some properties that have limited their applications in food fortification. Stigmasterol a plant sterol with several pharmacological activities is susceptible to oxidation when exposed to air, a process enhanced by heat and humidity. In this context, microencapsulation is a way of preventing oxidation, allowing stigmasterol to be incorporated into various pharmaceutical forms while increasing its absorption.^6-8^ Thus, microencapsulation technology has been applied to overcome some of the mentioned limitations. Encapsulation is a method which has been used in the form of micro- and nanoparticles for various food applications such as food protection to avoid adverse effects of some factors including heat, moisture, oxidation, formation of off-flavors, and evaporation or loss of volatile compounds.^9^

Polysaccharides and proteins are biopolymers which can join together through electrostatic attraction producing molecular complexes such as protein-protein, polysaccharide-polysaccharide, and polysaccharide-protein (nano) complexes.^10^ Protein-polysaccharide complexes can be formed by different approaches such as covalent bound formation through Maillard reaction or ionic interactions in polyelectrolyte complexes.^11,12^ These complex structures can be used to encapsulate hydrophobic and hydrophilic nutraceuticals such as vitamins, essential oils, fatty acids, flavors, etc.^13^ polysaccharide and protein interaction affects on functional and physicochemical characteristics of the final mixture including thermal stability, solubility, foaming ability, and surface tension.^14^ Two biopolymers that were used in this study were gelatin and chitosan. Gelatin is a protein which can be produced from the basic hydrolysisof collagen and can also be obtained from various sources such as fish (1.5%), cowhide (29.4%) pig skin (46%), and pig bone (23.1%). The surface charge of gelatin above its isoelectric point is negative while below it is positive.^15-17^ Chitosan is soluble at low pH values which can limit its function as an encapsulating compound. However, because of mucoadhesive properties of this polymer, it has been used frequently in controlled-released delivery systems. Further, the efficiency of microencapsulation method can be enhanced using a combination of two or more shell materials.^18-21^

Recently, there were more general attraction towards fabrication of nanofibers (NFs) from mixed biopolymers for nanoencapsulation of bioactive compounds using the electrospinning method.^22^ Electrospinning technique uses electrostatic force to produce fibers with diameters from micrometer to nanometer.^23^ The electrospinning procedure has various advantages such as the absence of heat and high encapsulation efficacy of bioactive constituents upon processing storage. NFs that are obtained from electrospinning process have advantages such as high porosity, large surface areas, low density, and small pore sizes.^24^ The spinnability of polymers can be affected by some factors including, viscosity, electrical conductivity, conformational shape, and surface tension of polymers.^25^ Generally, proteins are known as biopolymers which cannot be spun due to their complex secondary and tertiary structures and weak internal interactions.^26^ Thus, synthetic polymers including polyvinyl alcohol and polyethylene oxide can be used to increase their spinnability.^27^

To the best of our knowledge, there is no report published on fabrication of chitosan-gelatin electrospun fibers without adding synthetic polymers. Thus, in this study the spinnability of mixed chitosan and gelatin solutions was investigated at different polymer ratios, and the physicochemical properties of the fabricated electrospun fibers were evaluated.

## Materials and Methods

Stigmasterol (assay purity 97%) and medium molecular-weight chitosan with >75 % degree of deacetylation was purchased from Sigma Aldrich (USA). Gelatin (obtained from cold water fish skin) was bought from Sigma Chemical Co. (St. Louis, MO). Acetic acid was acquired from Merck Co. (Germany). For preparation of all solutions, deionized water was used.

### Preparation of target biopolymer solutions

To choose the optimum concentration of gelatin spinnability, the tests were accomplished at various concentrations of gelatin. Firstly, different concentrations (12-20 % w/v) of gelatin was solubilized in aqueous acetic acid (90% v/v) and stirred for 2 hours to hydrate it completely. In this study, the concentration of chitosan was considered constant. A chitosan solution (1.5 % (w/v)) was prepared in 90% v/v aqueous acetic acid for 2 hours under magnetic stirring at room temperature. In the next step, chitosan solution was added to the gelatin solutions at various ratios under stirring (Table 1). The final solutions were used for spinnability examination. The electrospun fiber obtained from the examination was placed in a 25°C vacuum oven for one day to remove any remaining solvent. The fabricated fiber was chosen according to results from scanning electron microscope (SEM) and spinnability examinations. For preparing stigmasterol-loaded NFs, the ethanolic solution of stigmasterol was added to the polymers’ solution. The concentration of stigmasterol in the mixture was set at 0.04% w/v.

**Table 1:** Physicochemical properties of chitosan-gelatin solutions^a^

**Chitosan (%w/v)**	**Gelatin (%w/v)**	**Ratio**	**Spinnability**	**Viscosity (mPa.s)**
1.5	20	100:0	**-**	109.00±2.83^d^
1.5	20	75:25	**-**	126.00±4.24^c^
1.5	20	50:50	**++**	140.00±1.41^b^
1.5	20	25:75	**++++**	154.00±2.82^a^

^a^Data reported are average values ± standard deviations. Values within each column with different letters are significantly different (*P* < 0.05)

### Electrospinning of biopolymer solutions

High voltage power of 17 kV with a flow rate of 0.2 mL h^−1^ was applied to the biopolymer solutions, and were then sucked up in a 10 ml plastic syringe attached to a blunt-tipped 21-gauge needle. In order to collect the NFs, an aluminum foil with a nozzle-collector distance of 15 cm was used. The electrospinning protocol was accomplished under ambient conditions at 25 ± 3°C and 30 ± 2% RH.

### Viscosity measurement of solutions

The viscosity of solutions was determined using Physica MCR 301 rheometer (Anton-Paar GmbH, Graz, Austria) after transferring 2 mL of the samples into cone and plate cylinder geometry with the shear rate range from 0.1 to 1000 s^−1^ at 25°C. (Measuring System: CP50-1-SN14598; d=0.099 mm). The viscosity of biopolymer solutions was identified at 100 s^−1^.

### Antioxidant activity of chitosan and chitosan-gelatin NFs

Antioxidant activities ofchitosan and chitosan-gelatin NFs, in the presence of stigmasterol or without it, were evaluated by 2,2-diphenyl-picrylhydrazyl (DPPH) radical scavenging analysis as defined previously with minor changes.^28^ Briefly, 40 mg of each sample was dissolved in 2 mL ethanol by consistently stirring at 200 rpm/min. After adding 2 mL (0.01mM) of DPPH solution into the test tube, it was incubated for 50 min, and then the solutions were centrifuged at 4000 × g (Hettich ROTFIX 32A). The absorbance of the supernatant was determined at the wavelength of 517 nm using a spectrophotometer (Perkin Elmer, Lambda2).

$$Scavenging\;effect\left(\%\right)=\frac{Blank\;absorbance\text{​}-Sample\;absorbance}{Blank\;absorbance\text{​}}*100.$$

### Encapsulation efficiency

In order to determine the encapsulation efficiency (EE %), 5 mg of stigmasterol-loaded fibers was taken in 1 mL of ethanol and then the mixture was centrifuged at 4000 rpm for 6 minutes at ambient temperature to remove any stigmasterol attached on the surface of the encapsulated structure. Further, 2.5 mL of ethanol was added to the precipitate from the centrifuge and the mixture was centrifuged again at 8000 rpm for 8 minutes. The supernatant was then used for HPLC injection. HPLC-UV analysis was conducted on a high performance liquid chromatography apparatus (Cecil CE-4900, Cambridge, England) consisting of multiple solvent delivery units, two CE-4100 pumps, mixing chamber, six-port valve (Rheodyne, USA), vacuum degasser, and CE-4200 i=UV–Vis detector (Cambridge, England). The used column for separation was an analytical C18 column (4.6 mm ID × 25 cm, 5 µm particle diameter) (Knauer, Germany) and the analysis was done at room temperature. The mobile phase was 20:80 (V/V) ratio of methanol and phosphate buffer solution (10 mM) with the pH of 6.8 set for the final solution. The method was isocratic and the flow rate was 1 mL/min. The stigmasterol quantification was carried out at 346 nm, and the calibration curve was plotted as wavelength versus 1-30 ppm concentrations of stigmasterol in ethanol. The EE was calculated using the following equation:

$$EE\%=\frac{\mathrm{The}\;\mathrm{amount}\;\mathrm{of}\;\mathrm{stigmasterol}\;\mathrm{in}\;\mathrm{supernatant}}{\mathrm{Total}\;\mathrm{amount}\;\mathrm{of}\;\mathrm{stigmasterol}\;}*100.$$

### Scanning electron microscopy (SEM) analysis

Firstly, the samples were sputtered with a gold–palladium mixture in vacuum and then the structure and morphology of NFs were evaluated by SEM (HITACHI model SU3500). The acceleration voltage of 25 kV was applied. The distribution of the diameters of fibers was determined through randomly measuring the diameter of approximately 100 fibers from SEM images at magnification of 5000×, using Image J software.

### Fourier transform-infrared spectrometry (FT-IR) analysis

Chitosan and gelatin powders andelectrospinning fibers were pressed with KBr pellets and then FT-IR analysis was conducted using a spectrometer (Agilent Technologies Cary 630 FTIR). Interferograms were collected over a spectral range of 4000–500 cm^−1^ with a nominal resolution of 2 cm^−1^ and 100 scans.

### Thermo gravimetric analysis (TGA)

Thermogravimetric analyzer (TGA 50, Shimadzu) was used for TGA and calcium oxalate monohydrate was used for apparatus calibration. Approximately 15 mg of samples was heated from 25 to 600°C at a scanning rate of 10°C per minute under nitrogen atmosphere.

***In vitro*** **release**

Dialysis bag diffusion technique was used for evaluating the *in-vitro* cumulative release of stigmasterol from the gelatin-chitosan NFs (cut off value =11000 Da) by imitating oral delivery to a specific site in the small intestine, where the NFs transit gastric condition is pH 1.2 for 2 hours in stomach, and intestinal condition is pH 6.8 for 3 hours in small intestine.^29^ To imitate stomach environment with and without enzyme, 1 mL of the delivery system containing stigmasterol was added into the dialysis bag and placed in 20 mL of HCl (0.1 M)-ethanol w/v (80:20 ratio) in the presence and absence of pepsin. Then, the dialysis bag was put in 20 mL phosphate buffer-ethanol (80:20 ratio) under magnetic stirring (50 rpm and 37°C) to simulate small intestine environment. The stigmasterol amount was determined at the end of simulation time.

## Results and Discussion

### Electrospinning and physicochemical properties of chitosan-gelatin solutions (characterization of NFs)

**Figure 1:**
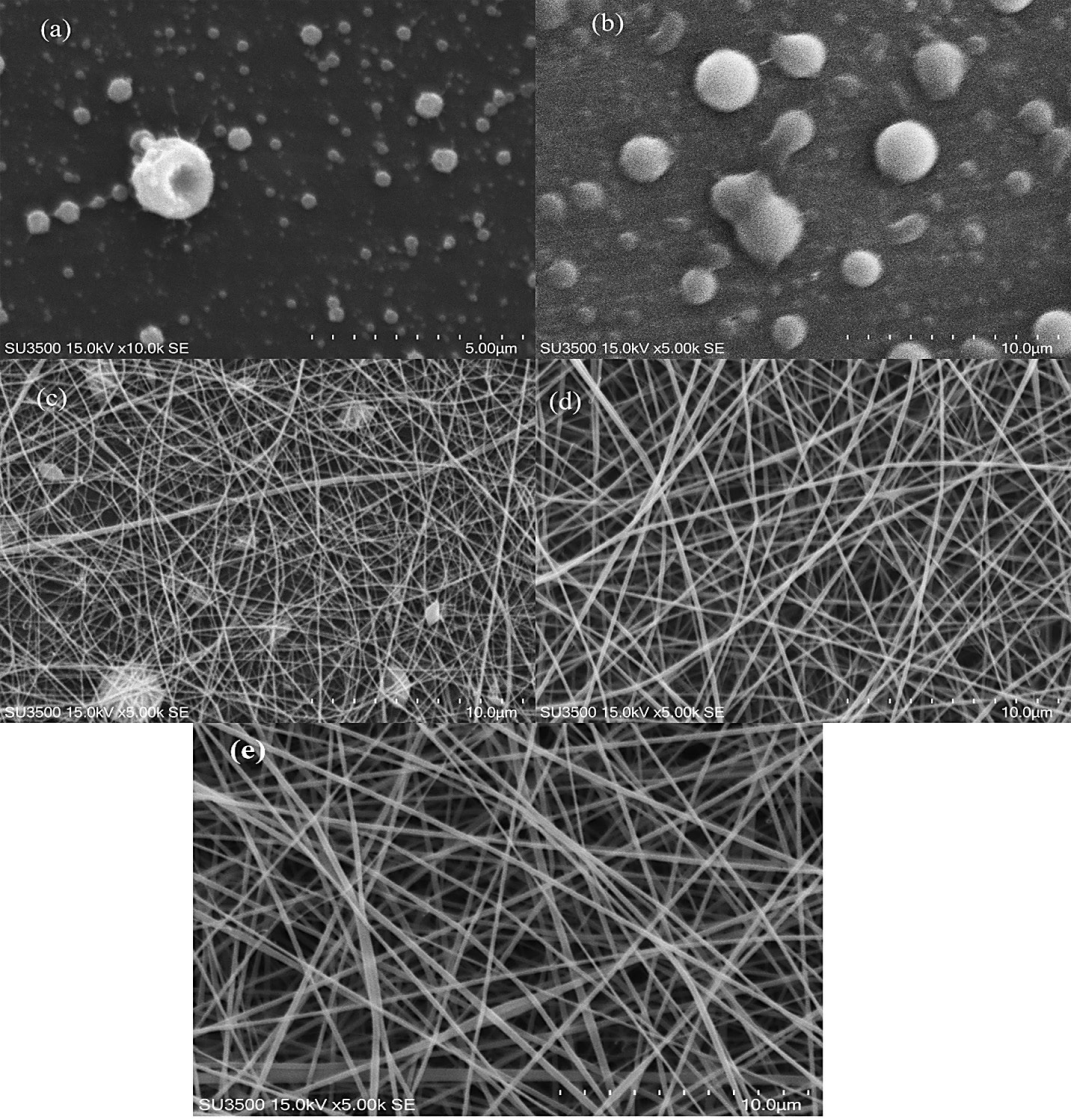
SEM image of chitosan-gelatin nanofibers (NFs) (1.5-20%w/v) at ratio of 100:0 (a), 75:25(b), 50:50 (c), 25:75(d) and stigmasterol loaded 25:75 at 0.04 %w/v (e).

The spun formation capability of the polymer solutions was primarily evaluated through visual observation. SEM images of samples were used to determine the diameter and morphology of fibers. SEM images of chitosan and gelatin solutions at various ratios are shown at Figure 1. According to the images, the quality as well as the diameters of chitosan fibers in acetic acid increased with gelation concentration rise, where the fibers formed with higher concentrations of gelatin (20 % w/v) were more uniform and they had lower amount of bead in them (Figure 1d). A solution of 1.5% chitosan alone as well as gelatin-chitosan solution at the ratio of 3 to 1 could not form a NFs, but with the decrease in the amount of chitosan and increase in gelatin concentration, uniform fibers were formed (Figure 1c and 1d). Fibers were composed of 50: 50 ratios of chitosan-gelatin containing beads Figure 1c. The best and most uniform fiber was formed at the chitosan-gelatin ratio of 1 to 3, Figure 1d.

According to the former studies that used acetic acid as the solvent for the formation of chitosan fibers, spark was formed during electrospinning procedure which was due to the high conductivity of acetic acid and its effect on the viscosity of chitosan solution.^30^ Gelatin addition to chitosan solution had a positive effect on the electrospinning protocol and the quality of NFs. As mentioned in previous studies, the physicochemical properties of biopolymer solutions such as viscosity have a significant role in the formation of uniform and continuous fibers.^25,31^ The viscosity of chitosan solution increased in the presence of gelatin (Table 1). Generally, it can be realized (Table 1) that increase in the amount of the protein polymer in the mixture solutions causes an increase in the apparent viscosity (thus it can be approved that the addition of gelatin improves chain entanglement for fiber formation). As shown in SEM images (Figure 1c and 1e), by increasing the apparent viscosity, bead free and more uniform fibers were formed at the ratio of 25:75 of chitosan-gelatin solutions.

**Figure 2:**
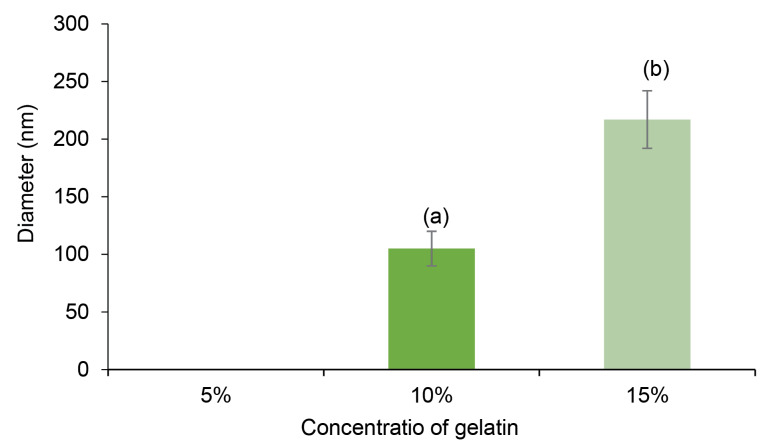
Diameter of fibers of chitosan–gelatin. (a): 50-50 chitosan–gelatin, (b): 25-75 chitosan–gelatin. At concentertion 5% of gelatin the fibers were not formed.

The measured diameters of fibers in the chitosan-gelatin at the ratio of 75:25 were zero (the fibers were not formed), the mean diameter of fibers at the ratio of 50:50 were 105 ± 35 nm, while the diameter of fibers at ratio of 25:75 (chitosan-gelatin) meaningfully increased to 217 ± 43 nm, which was comparable to 15 % w/v gelatin in formulation (Figure 2). As observed in Figure 1d and 1e, increase in the concentration ratio of gelatin in the blend (25:75 of chitosan-gelatin) has a positive effect on the morphology of fibers and the spinnability of biopolymer solution.

According to the previous investigations in electrospinning method, the viscosity and surface tension of solutions play an important role in the protocol control.^32^ The surface tension determines the spinnability of polymer when the viscosity of polymer is low; however, in the case of a viscose biopolymer solution such as 20 % w/v gelatin, the viscosity is a factor that controls the productivity of electrospinning. Moreover, gelatin implicitly reduces the conductivity of biopolymer solutions, which limits spark creation during electrospinning process. There are limited studies on the electrospinning of mixed polymers (chitosan and agarose – trifluoroacetic acid solvent, chitosan, and gellan-trifluoroacetic acid solvent).^33-35^ The obtained result of this study about spinnability is in agreement with other studies in which the spinnability of chitosan increased in the presence of agarose and gellan. Thus, electrospinning productivity improved with optimum values of conductivity and chitosan-gelatin solution’s viscosity, which was attained at a defined concentration ratio. Addition of stigmasterol to chitosan-gelatin solution (at the ratio of 25:75) resulted in more suitable formulations according to SEM and spinnability results. The SEM image of 0.04 % w/v stigmasterol loaded NF is displayed in Figure 1e. Uniform fibers without any beads were produced with certain diameters 208 ± 32 nm. The increase in stigmasterol level up to more than 0.04% w/v revealed a negative effect on fibers’ morphology and electrospinning yield.

### Antioxidant activity

**Figure 3:**
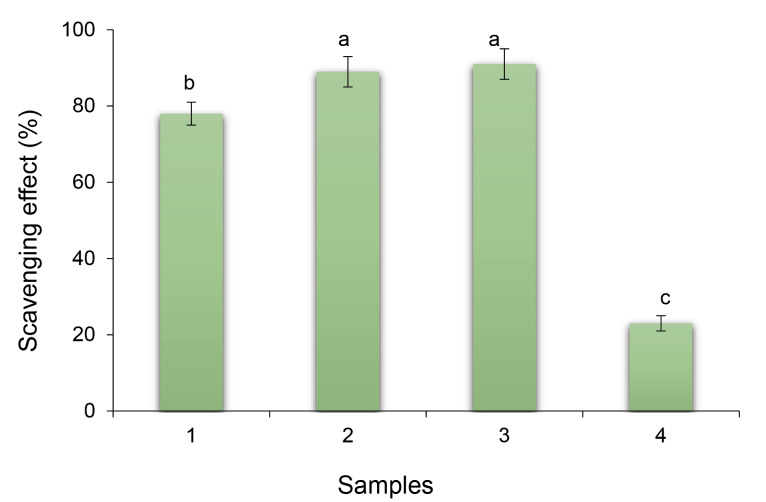
Antioxidant activity of stigmasterol loaded nanofibers (NFs) against free stigmasterol and gelatin NFs. 1) Stigmasterol (40 uM), 2) Gelatin-stigmassterol NFs, 3) Chitosan-gelatin-stigmasterol NFs, 4) Gelatin NFs. Different letters in each column represent significant differences (*P* < 0.05).

The antioxidant activity results indicated that addition of stigmasterol significantly increased the antioxidant activity of NFs (Figure 3). According to earlier investigations, a growth in surface area, specifically in NFs, can cause increase in antioxidant capacity of bioactive components.^36^ Furthermore, due to the active amino and hydroxyl groups in the chitosan, the polymer chains show antioxidant activity, which are the reasons that chitosan has hydrogen-donating ability.

### Encapsulation efficiency

**Figure 4:**
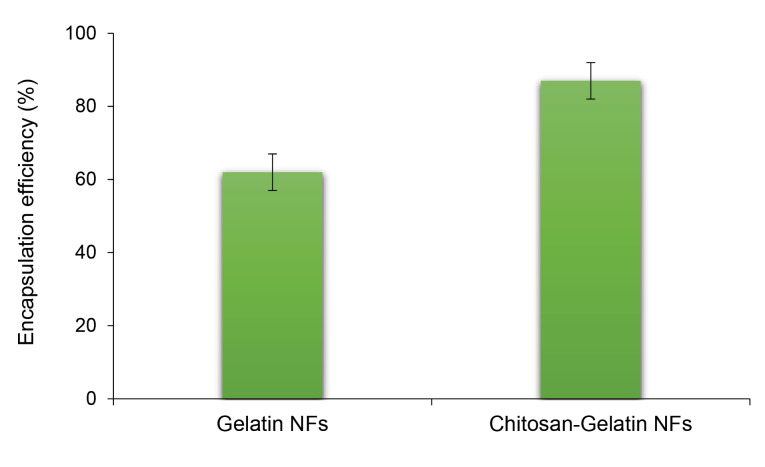
Encapsulation efficiency of stigmaserol in gelatin NFs and chitosan-gelatin NFs (at ratio of 25:75).

EE of stigmasterol in gelatin fibers was 62 ± 4%, but it increased to 87 ± 5% in fibers formulated using chitosan-gelatin at the ratio of 25:75 (Figure 4). The chitosan has a significant role in improving the quantity of physical entrapment of stigmasterol during electrospinning. During EE measurement, the physical form of the electrospun fibers was determined. After adding the fibers to the ethanolic solution the fragmented to visible particles were obvious in the solution, and after centrifugation step the undissolved particles could be seen in the bottom of the tube.

### FT-IR

**Figure 5:**
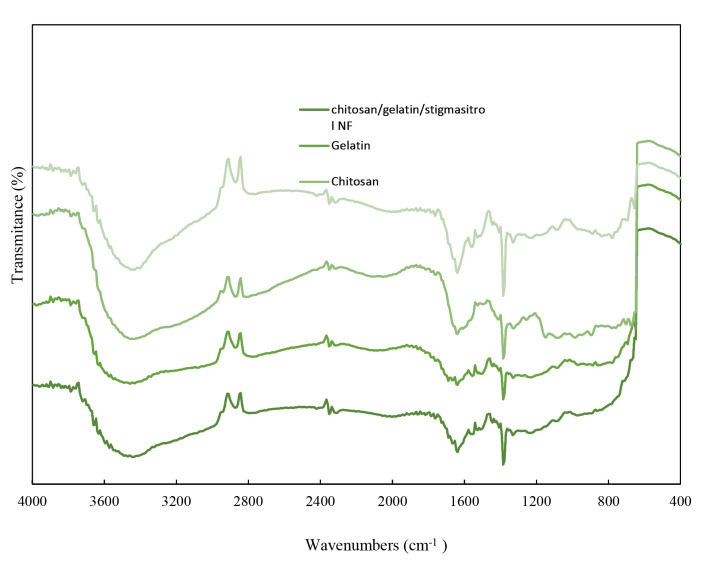
Fourier transform infrared spectroscopy (FT-IR) spectra of chitosan,gelatin, Stigmasterol-loaded chitosan-gelatinNFs and chitosan-gelatin NFs. (a) Chitosan-Gelatin-Stigmasterol NFs, (b) Gelatin, (c) Chitosan, (d) Chitosan-Gelatin NFs.

The FT-IR spectra of pure and chitosan NFs are shown in Figure 5. In all spectra, the absorption peak appeared at around 3400 cm^−1^ and 1629 cm^−1^ associated with the asymmetric and symmetric stretching vibrations of the hydroxyl group (O-H). The patterns related to the gelatin structure and vibrations of C=O and N-H bonds in amide I, as well as vibrations of C≡N and N-H groups in amides II and III, also appeared at around 1633 cm-^1^, 1583 cm-^1^, and 1238 cm-^1^.^35^ The peaks which appeared in 1518 cm-^1^ were attributed to the vibration mode of the N-H band in amide II, and the peak appearing at shorter wavelengths indicated N-H vibration of NH_3_ group in the chitosan structure.^37^ The change in the intensity and location of the peaks is 5 to 10 cm-^1^, which is an evidence for the presence of both materials and the successful preparation of the two-component NFs.

### TGA analysis

**Figure 6:**
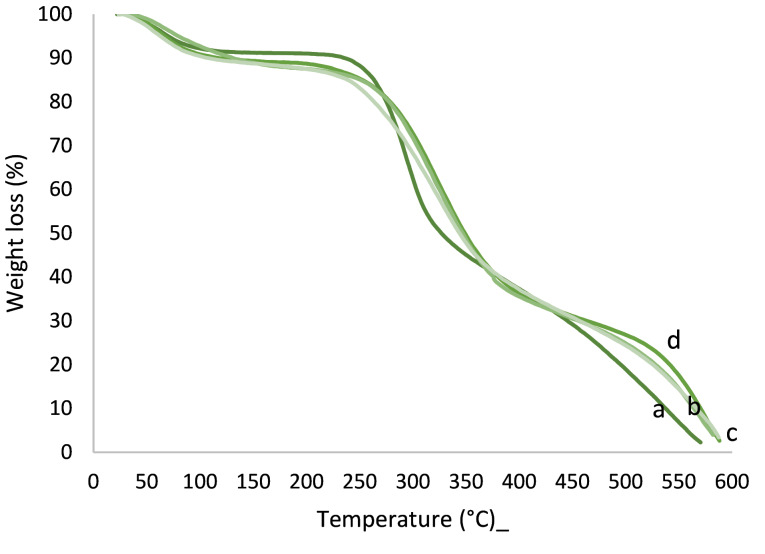
Thermogravimetric spectra of (a) chitosan, (b) chitosan-gelatin-stigmasterol, (c) gelatin, (d) chitosan-gelatin NFs.

TGA graphs of chitosan, gelatin, and their two-component NFs are shown in Figure 6. As can be seen, less weight loss occurred at around 70°C for all samples and more weight loss occurred at the temperatures of around 180°C and 300°C, respectively, which are related to the removal of adsorbed water, deacetylation, and decomposition of chitosan above its melting point. At 600°C, all samples are completely decomposed. Note that the stability of NFs increased due to the addition of gelatin to chitosan.

### Release behavior in gastrointestinal

**Figure 7:**
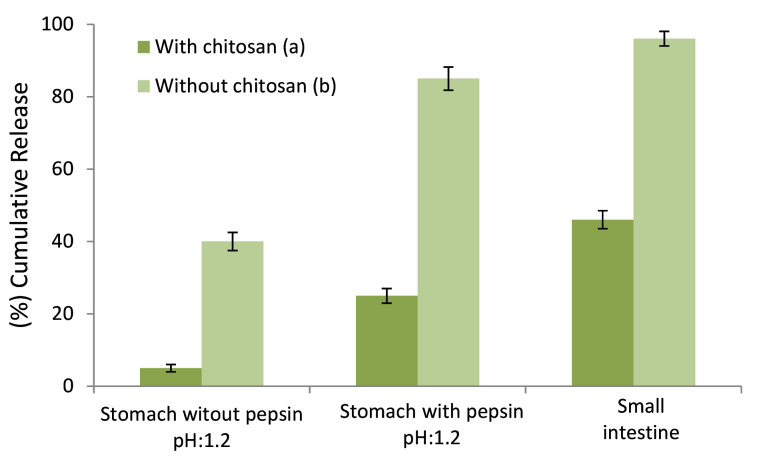
Cumulative release behavior of stigmasterol loaded NPs in mimic of gastroin-testinal conditions. (a) with chitosan, (b) without chitosan.

Figure 7 depicts the release behavior of stigmasterol from gelatin-chitosan colloidal NFs. The amounts of stigmasterol release in small intestine in the presence of chitosan formula were significantly less than those without chitosan formula (46 ± 3 % and 96 ± 4 % respectively). Note that the contents of stigmasterol delivered to small intestine in formulations with and without chitosan were between 71 ± 3 % and 10 ± 5 % of the initial load of stigmasterol. Thus, this newly colloidal system (gelatin-chitosan colloidal NFs) has a promising potential for small intestine target delivery of stigmasterol.

In each of the stigmasterol loaded systems, in the presence of pepsin, the release at pH 6.8 was quicker than that of pH 1.2. The mechanism of pH-sensitive release is that amine groups of chitosan are being protonated at low pH, and hydroxyl groups of gelatin are being deprotonated at high pH. In the presence of pepsin at pH 1.2, the release was far faster than at pH 6.8 which might be due to the protease property of pepsin.

## Conclusion

In this study, stigmasterol loaded food-grade NFs with aqueous solutions of chitosan-gelatin were successfully fabricated using electrospinning method without using any synthetic polymers. Ultra-thin beads free NFs were prepared with a high concentration of gelatin (20% w/v) and chitosan (1.5 % w/v) at 25:75 (chitosan-gelatin) and 0.04 % w/v stigmasterol. Electrospinning productivity was enhanced upon the addition of gelatin. The concentration of polymeric solution and viscosity had a notable effect on the electrospinning efficiency of the chitosan-gelatin-based NFs.The results of this study suggested that gelatin has a high potential for enhancing the spinnability of chitosan under acidic conditions at optimized concentrations.

## Ethical Issue

Not applicable

## Conflict of Interest

The authors have declared that there is no conflict of interest.

## Acknowledgments

The authors gratefully acknowledge Miss. Mohammadian (PhD candidate of physical chemistry in the Shahid Beheshti University) for her help in the analysis and characterization of the samples. Furthermore, this research was a part of PhD dissertation at Shahid Beheshti University of Medical Sciences, Tehran, Iran.
